# Stabilizing nanolasers via polarization lifetime tuning

**DOI:** 10.1038/s41598-021-97757-8

**Published:** 2021-09-17

**Authors:** Aycke Roos, Stefan Meinecke, Kathy Lüdge

**Affiliations:** Institute for Theoretical Physics, Technische Universiät Berlin, Hardenbergstr. 36, 10623 Berlin, Germany

**Keywords:** Nanophotonics and plasmonics, Optical physics, Physics, Statistical physics, thermodynamics and nonlinear dynamics, Nonlinear phenomena

## Abstract

We investigate the emission dynamics of mutually coupled nanolasers and predict ways to optimize their stability, i.e., maximize their locking range. We find that tuning the cavity lifetime to the same order of magnitude as the dephasing time of the microscopic polarization yields optimal operation conditions, which allow for wider tuning ranges than usually observed in conventional semiconductor lasers. The lasers are modeled by Maxwell–Bloch type class-C equations. For our analysis, we analytically determine the steady state solutions, analyze the symmetries of the system and numerically characterize the emission dynamics via the underlying bifurcation structure. The polarization lifetime is found to be a crucial parameter, which impacts the observed dynamics in the parameter space spanned by frequency detuning, coupling strength and coupling phase.

## Introduction

Coupled nanophotonic semiconductor lasers are a prototypical model for on-chip laser networks^1,2^, which have attracted considerable attention as an optical solution for neuromorphic realizations in the recent years^3–5^. Due to their small footprint, high speed and low power consumption, they are promising light sources for a wide range of nanophotonic applications such as photonic integrated circuits, on-chip optical computing, and optical communication^4,6–9^. One crucial precondition for a successful photonic implementation is the knowledge of the synchronization stability boundaries and thus the underlying dynamics of the devices.

Micro- and nanolasers using metal cavities^10^ or made of 2D material^11^ differ from conventional meso- or macrolasers by their high photon loss rates^12–15^. The effect of a high spontaneous emission rate leads to interesting stochastic effects close to threshold^13–16^, but also leads to an increased dynamical complexity of such lasers. Their deterministic dynamics are well described by macroscopic Maxwell–Bloch equations, which are structurally aquivalent to the Lorenz-Haken equations^17–20^. In particular, the dynamical influence of the polarization of the active medium can not be neglected for such lasers. Previous works have shown that this additional degrees of freedom can have a stabilizing effect onto the dynamics in injection and feedback setups^12,21^. We want to deepen this analysis and predict optimal configurations for delay coupled nanolasers with a focus on frequency tuning and locking ranges. The nanolasers investigated here are assumed to be single mode in order to emphasize the effect of the internal timescales. Therefore, dynamic effects induced by the gain competition of two-modes, as e.g. discussed in^22–24^, are omitted for clarity.

The dynamic classification of lasers into class-A, -B and -C is determined by the effective number of dynamical degrees of freedom^25,26^. Class-A lasers are characterized by a long photon lifetime in the cavity while the inversion and polarization lifetime are much smaller, thus, the electric field is the dominating dynamical quantity. For a class-B laser the inversion lifetime matches the photon lifetime and is therefore treated as an additional dynamic variable. Lastly, for a class-C laser, all three timescales are on the same order of magnitude and thus the dynamic degree of freedom of the polarization also plays a crucial role.

A nanolaser with its short cavity lifetime is usually a class-C laser^27,28^. In this paper, we do not want to provide a quantitave modelling of nanolasers, as e.g. possible in more elaborate frameworks^29^, but we will discuss the dynamic effect of the timescales. In particular, we explore the impact of the polarization lifetime onto the locking behavior of two coupled nanolasers. We use a paradigmatic (Maxwell–Bloch) class-C model and discuss the bifurcation structure with a special focus on the systems symmetries. We further show that polarization lifetimes on the order of the photon lifetime yield optimal locking behavior over wide tuning ranges. Our results are important for applications where synchronization among coupled lasers is crucial and where a wider tuning range allows for a higher tolerance towards volatility in the fabrication process.

Since meso-/macroscopic semiconductor lasers are proven to be described well by class-B lasers, a wide range of investigations has been carried out over the recent years. Therefore comprehensive insights on several setups as lasers with optical injection or feedback^30–32^ and coupled laser systems^7,33–37^ have been gathered. For class-C lasers it was theoretically predicted that injection and feedback setups show significant differences^12,21^. Here, we investigate the dynamics of two identical coupled class-C lasers. Since it is experimentally almost impossible to realize two identical lasers, we analyze the lasers under small frequency detunings, which measure the effects of small differences between the lasers. We further study the system under a transition of the polarization lifetime from vanishing (class-B) to large times (class-C) to connect this manuscript to literature concerning coupled semiconductor class-B lasers^33–36,38^.

The paper is structured as follows: In “Semi-classical laser model” and “Symmetries” sections we introduce the model of two coupled class-C lasers, derive a class-B model as limit of small polarization lifetimes and discuss the symmetries of the coupled laser system. In “Compound laser modes (CLMs)” section we investigate the steady-state solutions (compound laser modes) with an emphasis on the polarization lifetime and the frequency detuning of the lasers. Finally, in “Bifurcation scenarios” and “Locking range” sections we discuss the bifurcation structure and study the maximum detuning (locking range) for which the lasers phase lock.

## Semi-classical laser model

**Figure 1:**
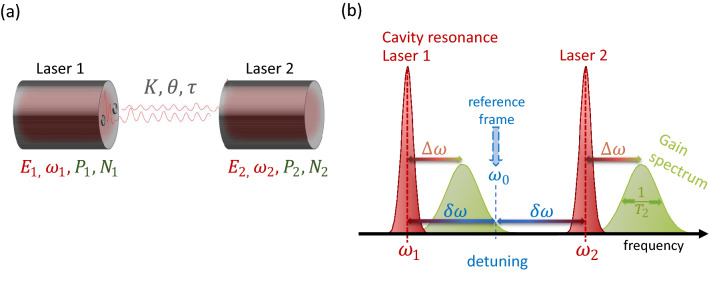
(**a**) Sketch of the coupling scheme and relevant dynamical variables and parameters. (**b**) Frequency scheme of the uncoupled lasers. The cold cavity frequencies $\omega _{1,2}$ of the lasers are detuned by $2\delta \omega$. For both lasers there is a shift $\Delta \omega$ between cavity resonance and gain maximum. The gain width is proportional to $\frac{1}{T_2}$ for $T_2>1$ (figure not to scale).

Lasers generally consist of a cavity containing an active medium that is constantly being stimulated by an external pump current *p* supplying the laser system with energy. The resulting inversion *N* of the medium induces an electric field *E*, which retrospectively causes a polarization *P* of the active medium. The modelling is done with macroscopic Maxwell–Bloch equations, both for atomic and semiconductor lasers^20^. They can be derived by a quantum-mechanical treatment of the active medium and a classical treatment of the electric field. The resulting microscopic Maxwell–Bloch equations can be transformed to macroscopic equations^18^. To describe two coupled lasers as shown in Fig. 1a, six differential equations, comprising the complex electric field E, the carrier inversion N and the complex polarization P for both lasers, are used. For a single laser we use the model published in^12^. The equations read

1a $$\begin{aligned} \dot{E}_{j}&= c P_j-\frac{1}{2}E_j+ (-1)^j i\delta \omega E_j+K e^{i\theta } E_k(t-\tau ) \end{aligned}$$

1b $$\begin{aligned} \dot{P}_{j}&= \frac{1}{T_2}[(i \Delta \omega -1)P_j+E_j N_j] + (-1)^j i\delta \omega P_j \end{aligned}$$

1c $$\begin{aligned} \dot{N}_{j}&= \frac{1}{T}[p-N_j-2c {\text {Re}}{(P_j E_j^{*})}] \end{aligned}$$

for $j,k \in \{1,2\}$ and $j\ne k$, where all time scales, such as the carrier lifetime *T* and the polarization lifetime $T_2$ are normalized to the photon lifetime in the cavity. To translate the timescales into *SI*-units, e.g., a photon lifetime of $T_{ph}=1\text {ps}$ can be assumed^39^. The pump current is denoted by *p*. The parameter

2 $$\begin{aligned} c=\left( \frac{2\Delta \omega }{T_2+2}\right) ^2+1 \end{aligned}$$

normalizes the system such that the first laser threshold is always at $p_{thr}^{(1)}=1/2$. Thus, it does not depend on the polarization lifetime $T_2$, which allows for a fair comparison^12^. Throughout this paper, we choose a pumping of $p=2$, which corresponds to the lasers operating four times above threshold and below the second threshold^12^. The parameter $\Delta \omega$ is the difference between the transition frequency of the two energy levels and the cavity mode. The system is further transformed into the reference frame of the average optical frequency $\omega _0$ of the two solitary laser frequencies $\omega _1$ and $\omega _2$. Thus, a frequency shift of $\delta \omega = (\omega _2 - \omega _1)/2$ with respect to the solitary laser equations is added to account for the detuning of the lasers. All frequencies described above are schematically shown in Fig. 1b. The coupling among the lasers is implemented via the term $K e^{i\theta } E_k(t-\tau )$ in Eq. (1a), where self-coupling is not considered. $\tau$ represents the propagation time between the lasers. We only consider a delay of $\tau =10$ which corresponds to a length of $800 \;\upmu \text {m}$ on an integrated chip. *K* is the coupling strength. In this manuscript we mainly choose a coupling of $K=0.1$, which refers to an intensity reflectivity of $20\%$. We consider superposition effects by back reflected light as small at this value and do not expect a significant impact on the observed dynamics^40^. The coupling phase $\theta$ is an additional phase shift acquired by the in-coupled light.

**Figure 2:**
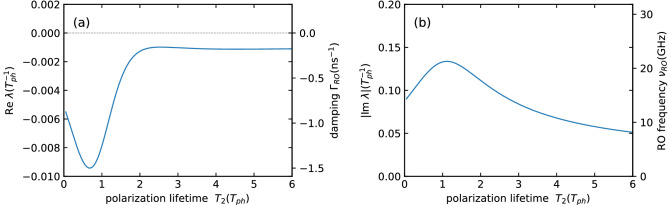
Complex eigenvalue of the linearized Class-C equations for a solitary laser. (**a**) Real part eigenvalue describing the relaxation-oscillation (RO) damping, (**b**) Absolute value of the imaginary part yielding the RO frequency as a function of the polarization lifetime $T_2$. The secondary y-axes show a conversion to physical units assuming a photon lifetime of $T_{ph}=1\text {ps}$. Other parameters are $\Delta \omega =3$, $p=2$, $K=0$ and $T=392$.

The complex eigenvalues of the linearized Class-C equations for one solitary laser have been determined as a function of the polarization lifetime $T_2$ for the parameters discussed throughout the manuscript. Without the delayed coupling there are in total five eigenvalues out of which one is zero due to the phase symmetry^12^. The real and imaginary part of the (negative) eigenvalue which is undamped first (the one with the smallest absolute real part) are plotted in Fig. 2. The real part Re $\lambda$ corresponds to the damping $\Gamma _{RO}$ of the relaxation oscillations, whereas the imaginary part Im $\lambda$ yields the relaxation-oscillation frequency $\nu _{RO}$.

The class-C laser equations can be reduced to class-B equations by considering the limit $T_2 \rightarrow 0$, which mimicks the transition from a nanoscale to a mesoscale laser. By carrying out an adiabatic elimination of the polarization *P* and setting the derivative of the polarization to zero, we obtain the class-B laser equations^12,41^

3 $$\begin{aligned} \dot{E_j} &= \left[(1 + i \Delta \omega) N_j - \frac{1}{2} \right] E_j + (-1)^j i\delta \omega E_j+K e^{i\theta} E_k(t-\tau) \\ \dot{N_j} &= \frac{1}{T}[p-N_j-2 N_j |E_j|^2] \end{aligned}$$

Note that the frequency difference between gain and cavity resonance $\Delta \omega$ now has an effect equivalent to the commonly known line-width enhancement factor $\alpha$^33,42,43^.

## Symmetries

Our model for the coupled laser system given in Eqs. (1a)–(1c) contains a number of symmetries that facilitate the interpretation of the results or enable the analysis of steady state solutions and the investigation of their stability. Hence, before presenting the results, the relevant symmetries are discussed in the following. These have been introduced by^33^ for two coupled class-B lasers and are reformulated and applied to class-C lasers here.

### Reflection symmetry

For identical lasers, Eqs. (1a)–(1c) are invariant under an exchange of the lasers. For non-identical lasers, i.e., here for a detuning $\delta \omega \ne 0$, the sign of the detuning has to be flipped

4 $$\begin{aligned} (E_1,P_1,N_1, E_2,P_2,N_2,\delta \omega ) \rightarrow (E_2,P_2,N_2, E_1,P_1,N_1,-\delta \omega ). \end{aligned}$$

### $2\pi$Translational symmetry

The system is invariant under $2\pi$-phase shifts of the coupling phase

5 $$\begin{aligned} (\theta ) \rightarrow (\theta + 2\pi n) \; \; \; \text {for } n \in \mathbb {Z}. \end{aligned}$$

### $\pi$-Translational symmetry

If the coupling phase $\theta$ is translated by $\pi$ this translation can be cancelled out by a $\pi$-phase shift of one of the laser fields

6 $$\begin{aligned} (E_2,P_2,\theta ) \rightarrow (e^{i\pi } E_2,e^{i\pi } P_2,\theta + \pi ). \end{aligned} $$

Note that the system satisfies more symmetries, which are however not crucial for the arguments within this manuscript.

## Compound laser modes (CLMs)

The fundamental steady-state solutions describe the asymptotic behavior of the coupled laser system. Such solutions have been previously discussed in the context of class-B lasers^33^ and are known as compound laser modes (CLMs). They describe fully synchronized, i.e., phase locked lasers. Due to the phase symmetry of the complex electric field and polarization we make the ansatz of constant light intensities $|E_j|^2$ and constant rotation in the complex plane.

7 $$\begin{aligned}  E_j = E_{j,0}e^{-i\Omega t + i\phi _{j,E}},~~ P_j = P_{j,0}e^{-i\Omega t + i\phi _{j,P}},~~ N_j = N_{j,0}. \end{aligned} $$

for $j \in \{1,2\}$. The electric fields given by the optical frequency $\Omega$ of the CLM are allowed to have a phase difference $\phi _{j,m}$ for $m\in \{E,P\}$. Inserting the ansatz into Eqs. (1a)–(1c) yields a non-linear system of 10 equations that exhibits several branches of CLMs, characterized by different properties. In the following sections, we restrict the analysis to CLM branches that contain stable CLMs and CLM branches, which interact with the former.

For identical lasers ($\delta \omega = 0$), the only CLMs found to be stable exhibit a phase difference between the lasers that is constant with respect to the coupling phase $\theta$. Therefore such solutions correspond to phase-locking, i.e., synchronization, among the lasers. We refer to those steady states, also known as symmetric solutions^35^, as constant-phase CLMs in analogy to^33^, where a similar model for two coupled class-B lasers is discussed. We define the phase shift as

8 $$\begin{aligned} \Delta \phi (\theta ) = \Delta \phi = \phi _{1,E}-\phi _{2,E} =\phi _{1,P}-\phi _{2,P}. \end{aligned}$$

**Figure 3:**
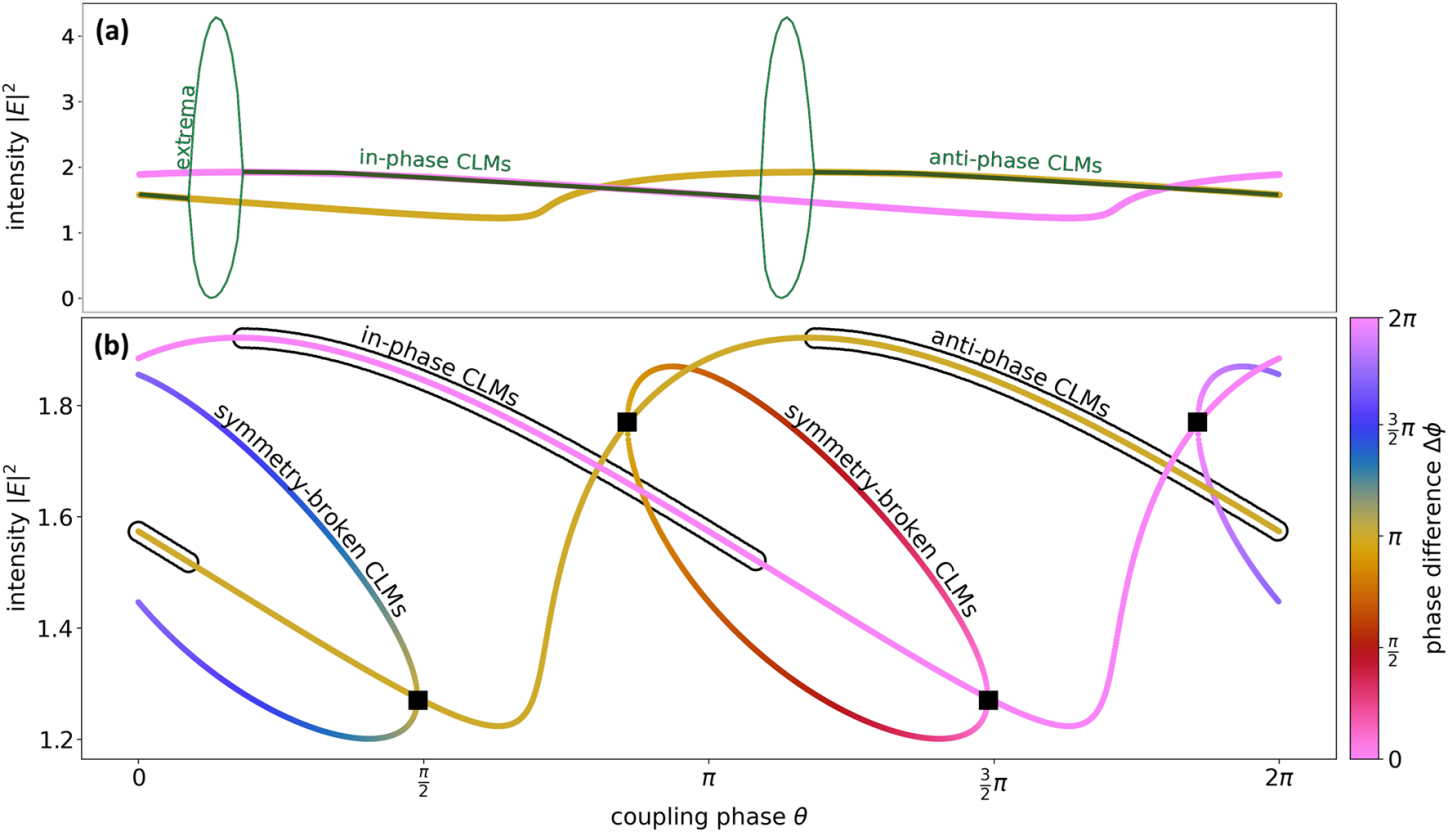
(**a**) Bifurcation scan of laser intensity $|E|^2$ as a function of the coupling phase (dark green, lasers are identical and emit the same intensity), yellow and pink lines show the constant phase CLMs, (**b**) intensity of constant-phase and symmetry-broken CLMs. The line color encodes the phase difference $\Delta \phi$ between the electric fields of the two lasers. The black squares denote pitchfork bifurcations^33^. Stable solutions in (**b**) are indicated by black surroundings. Other parameters are $T_2=2$, $\Delta \omega = 3$, $p=2$, $K=0.1$, $\tau =10$, $\delta \omega = 0$.

The amplitudes of the two lasers are identical when working on a constant-phase CLM. Figure 3a shows the intensities $|E_{j,0}|^2$ of the CLMs as a function of the coupling phase $\theta$. The color of the lines encodes the phase difference $\Delta \phi$ between the two lasers and accordingly emphasizes two branches of constant phase CLMs, the in- and anti-phase CLMs (pink and yellow) with a phase difference $\Delta \phi$ of 0 or $\pi$, respectively. The in- and anti-phase CLMs are connected by the $\pi$-translational symmetry: Given one full set of parameters, in particular $\theta =\theta _0$, for which an in-phase CLM exists, a corresponding anti-phase CLM exists for $\theta = \theta _0 + \pi$. Figure 3a also depicts the intensity extrema found via numeric integration in dark green. It can be seen that the lasers either emits on the CLMs or they show periodic intensity oscillations that are born in Hopf bifurcations (the oscillations are identical for the two identical lasers). These periodic dynamics exist in regions where the CLMs are unstable and the oscillations form bridges between the in- and the anti-phase CLMs (these bridges are also found for the class-B limit with long delay^36^ and for mutually delay-coupled semiconductor lasers in Ref.^38^ where they are called symmetric two-color states).

In Fig. 3b two additional branches of symmetry-broken CLMs (also named intermediate-phase CLMs in Ref.^33^, asymmetric solutions in Ref.^36^ or out-of-phase solutions in Ref.^35^) can be seen (curves that change their color) next to the constant phase CLMs. The symmetry broken CLMs are characterized by a phase difference $\Delta \phi (\theta )$ that varies with the coupling phase, and by non-equal amplitudes $E_{j,0}$^33,36,38,44^. As a result the solutions for the two lasers that operate on a symmetry-broken CLM, do not parametrize the same curves in $(\Omega ,N)$-space. As can be seen in Fig. 3b, the symmetry-broken CLMs emerge in pitchfork bifurcations (black squares) from either in- or anti phase CLMs and vanish in the other.

**Figure 4:**
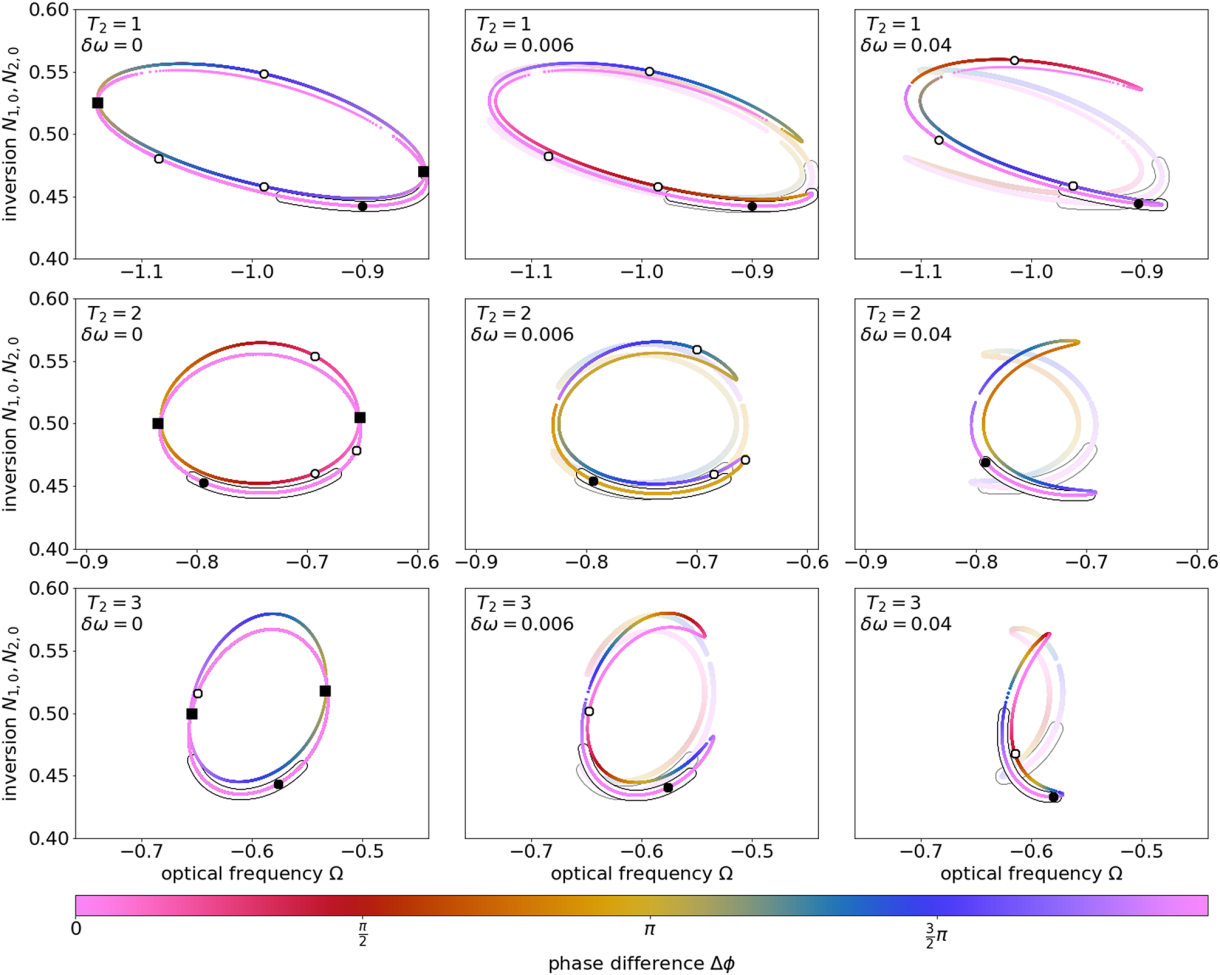
Compound laser modes (CLMs) depicted in $(\Omega ,N)$-space for $\theta \in \left[ 0,2\pi \right)$. From top to bottom the polarization lifetime $T_2$ increases. From left to right the detuning $\delta \omega$ increases. Constant-phase (pink lines) and symmetry-broken CLMs (lines with continuous color) can be distinguished by the color coded phase difference $\Delta \phi$. Pitchfork bifurcations are marked as black squares, solutions for $\theta = 0$ are indicated with white dots (black dots if stable). Stable solutions are indicated by black surroundings. For $\delta \omega > 0$, the two curves of CLMs connect to one that differs for each laser (transparent for laser 2). Other parameters are $\Delta \omega = 3$, $p=2$, $K=0.1$, $\tau =10$, $T=392$.

In analogy to the external cavity modes of lasers subject to feedback (steady state solutions of the intensity)^12,30^, Fig. 4 shows the CLMs in a $(\Omega , N)$-phase projection. Here a matrix of such projection-maps is shown, where the polarization lifetime increases from top to bottom (mimicking the transition from nano- to mesoscale lasers) and the detuning increases from left to right (i.e a change from identical to slightly different lasers). The in- and anti-phase CLMs can be found in pink and yellow in the left column for vanishing detuning. They lie on the same curves due to their identical amplitudes up to phase shifts of $\pi$. Stable solutions are indicated by black surroundings. Again the symmetry-broken CLMs (curves that change color in Fig. 4) connect the in- and anti-phase CLMs via pitchfork bifurcations (squares). Within every column of the matrix the polarization lifetime $T_2 \in \{ 1,2,3 \}$ is varied from top to bottom. This change in $T_2$ decreases the width of the gain spectrum, which is proportional to $\frac{1}{T_2}$ (see Fig. 1b). As a result, the solutions for the coupled laser system are found on a smaller range of CLM frequencies $\Omega$.

The consequences of non-vanishing detunings on the CLMs can be seen in the second and third column of Fig. 4. For each row, the polarization lifetime $T_2$ is constant and the detuning $\delta \omega$ is increased. Apart from $T_2$-dependent deformations of the curves, every row shows a similar trend: For non-zero detunings, the pitchfork bifurcations, where the constant-phase and symmetry-broken CLMs coincide, are destroyed and we observe an anti-crossing instead. The CLM branches link and form horseshoe formed closed curves. This happens for both lasers (laser 2 printed transparent). For the laser emission this means that both lasers now always emit at different intensities at the CLM solutions. Thus, due to the symmetry breaking induced by $\delta \omega$ (non-identical lasers) we loose the clear distinction between constant-phase and symmetry-broken solutions. The side of opening and closing of the horseshoe is opposed to each other for both lasers. The deformed horseshoe-curves include stable CLMs, marked by black surrounding. The curves shrink, which indicates that the lasers can not stabilize for arbitrarily large detunings. When increasing the detuning $\delta \omega$ we observe (not shown) that the horseshoe-formed curves pull together onto one point until they vanish completely and locking becomes impossible.

As mentioned before, the CLM solutions plotted in Fig. 4 are obtained by continuously tuning the coupling phase $\theta$. For the specific choice of $\theta =0$, the CLMs are marked by white and black dots. By inspecting those dots it becomes obvious that, for the fixed parameter set, there is only one stable CLM solution (black dot). This solution determines the emission frequency of the synchronized state observed numerically. It is noted that the size of the CLM curves increases with the coupling strength *K* (not seen in the figure). Their shape is not influenced by *K*, although the number of solutions, found for a fixed coupling phase, changes.

## Bifurcation scenarios

In order to analyze the synchronization behavior of the two coupled class-C lasers with respect to their coupling, we investigate the laser system with a focus on the coupling parameters $\theta$, *K* and $\delta \omega$. Two dimensional bifurcation analyses are carried out by integrating Eqs. (1a)–(1c) numerically, using random initial conditions for each data point.

**Figure 5:**
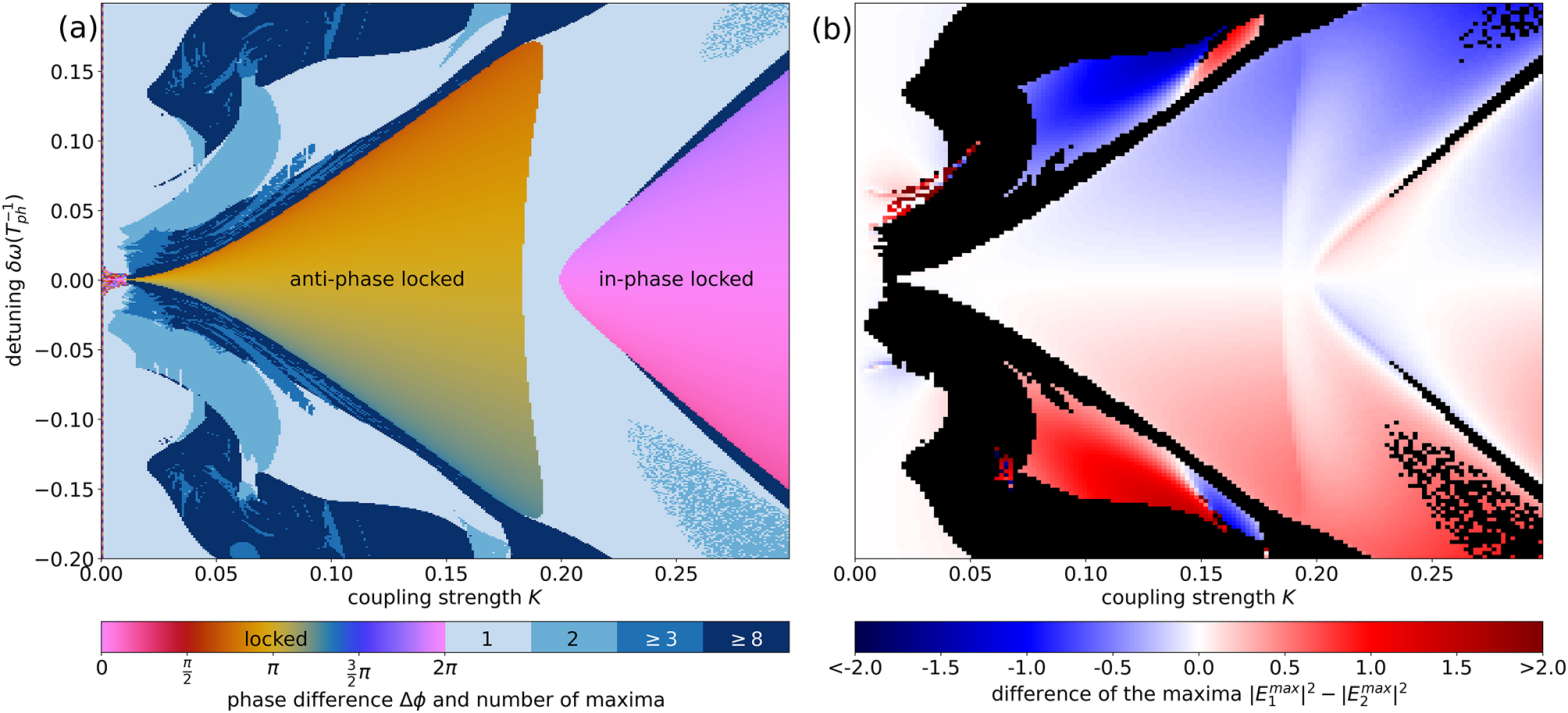
Dynamics in $(K,\delta \omega )$-space. (**a**) The number of maxima of time series $|E_1(t)|^2$ is shown in blue. The second color map encodes the phase difference $\Delta \phi$ between the electric fields of the two lasers when they are phase locked on a CLM. (**b**) Intensity difference between both lasers ($|E_1^{max}|^2-|E_2^{max}|^2$) color coded in blue and red, white indicates identical laser emission. Areas with more than one maximum are masked black. Other parameters are $T_2=1$, $\Delta \omega =3$, $p=2$, $\theta =0$, $\tau =10$, $T=392$.

Figure 5a shows a bifurcation diagram in the parameter space of coupling strength *K* and detuning $\delta \omega$. Every data point of the graph represents a time series of one of the lasers intensities $|E|^2$, where the number of maxima of this time series is evaluated. For zero maxima, the lasers settle onto a CLM (see “Compound laser modes (CLMs)” section). In this case, instead of the number of maxima, the plots show the phase difference $\Delta \phi$ of the electric fields. Figure 5b shows the difference $|E_1^{max}|^2-|E_2^{max}|^2$ of the maxima of the two lasers. We concentrate on the period-one and steady state emission and thus areas with two or more maxima are masked in black in (b). The symmetry-breaking of the CLMs caused by non-zero detuning $\delta \omega \ne 0$ can be seen by the red and blueish colored regions. In Fig.  4 we saw this detuning induced symmetry breaking via the different closed loop of solutions (different intensity for each laser). The white regions in Fig. 5b indicate symmetric operation of both lasers, which interestingly also occurs for non-vanishing detuning within the regions of period-one oscillations (light blue regions in Fig. 5a).

The overall bifurcation structure for the small value of $T_2=1$ chosen in Fig. 5, is in agreement with existing results on delay coupled class-B lasers. As expected for lasers with weak damping of their relaxation oscillations the locking range of the lasers approximately increases linearly with the coupling strength *K*. Please see Fig. 2 for the values of relaxation-oscillation frequency $\omega _{RO}$ and damping $\Gamma _{RO}$ of the solitary class-C laser used here.

For an increasing detuning, the locking cone is bounded by saddle-node bifurcations, while it collapses in a Hopf-bifurcation for increasing *K* around $K \approx 0.2$^35^. In Fig. 5 we can see the saddle-node characteristics as the dynamics changes abruptly from locked operation (yellow/pink) to complex dynamics (dark blue). Instead, at the Hopf bifurcation (vertical border of the locking range for increasing *K*) harmonic oscillations with one maximum (light blue colors in Fig. 5) are born (they can also be seen in the 1D-bifurcation scan in Fig. 3a). The bifurcation structure changes substantially with the coupling phase $\theta$ as can be seen in the animation (Supplementary Video S1), which shows graphs as in Fig. 5 with varying coupling phase $\theta$, where also the periodicity in the coupling phase $\theta$ (translations of $\pi$ or $2 \pi$) can be nicely seen. In particular, the $\pi$-periodicity can directly be associated with the $\pi$-translational symmetry of the in- and anti-phase CLMs. For phase differences of $\pi$ and $2\pi$, complex dynamics are observed for detuning $\delta \omega =0$ and small coupling strengths (Supplementary Video S1) which most likely emerge when the periodic bridges between in- and anti-phase dynamics form homoclinic connections^36^.

We note that the analysis of the coupled laser dynamics is performed for deterministic equations. Nevertheless, we checked if the dynamics is preserved when a Gaussian white noise term $D\xi$ is added onto the field equation. Our results show that as long as the noise strength *D* is chosen below $D\approx 0.1$ (corresponding to a relative standard deviation $\sigma /\langle I \rangle \approx 0.8$ for the solitary class-C laser), the locking structure is preserved. For $D> 0.1$ the noise dominates the dynamics.

The significant dependency of the locking behavior on the coupling phase $\theta$ suggests an analysis of the system with respect to this parameter. Within this projection we will also further investigate the impact of the polarization lifetime on the locking behavior of the lasers. Experimentally the coupling phase $\theta$ can easily be varied without crucially changing the other parameters. The normalized $T_2$-time can be changed either by tuning the photon lifetime, i.e., the cavity, or by adjusting the active medium.

**Figure 6:**
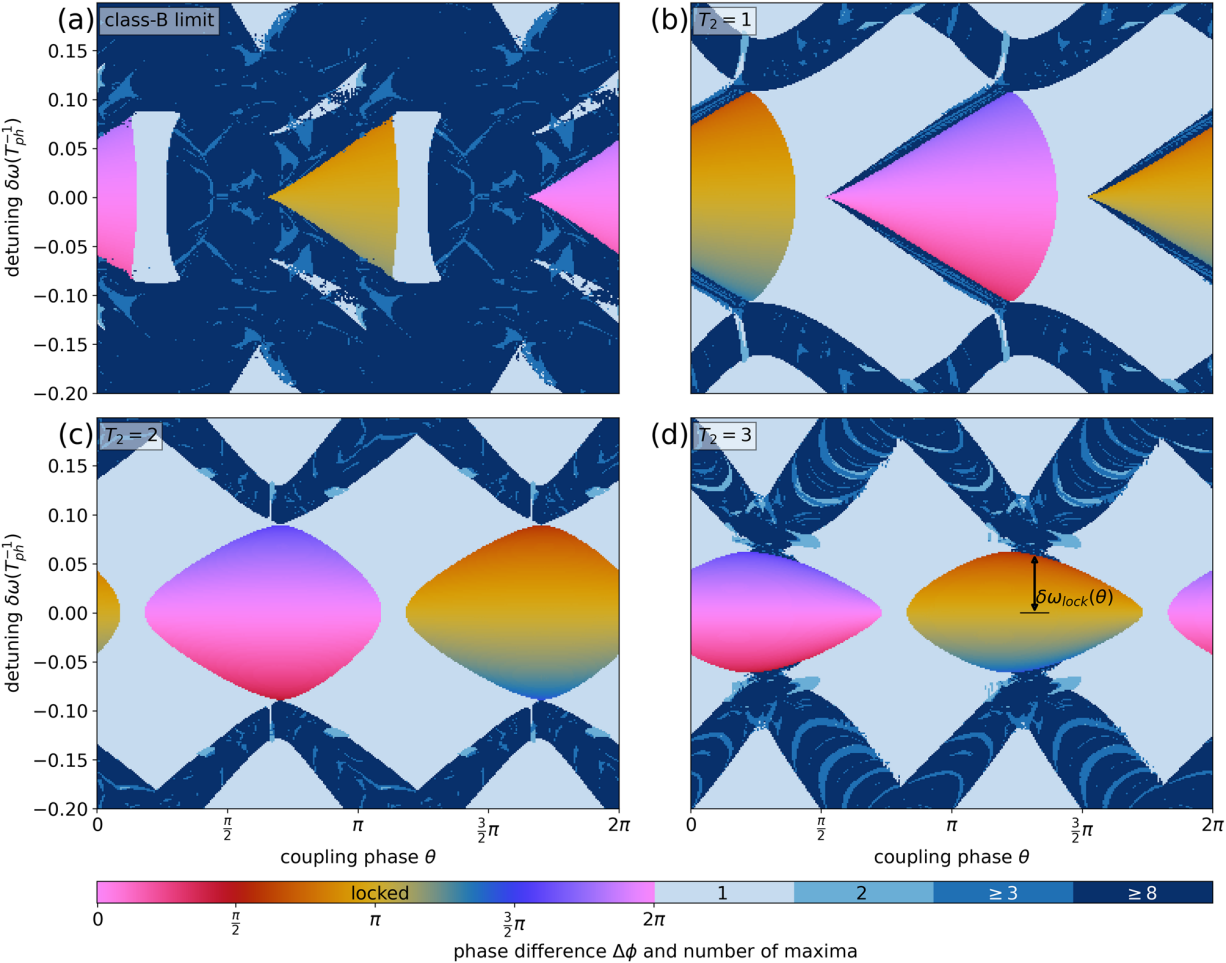
Two dimensional bifurcation scenarios in the $(\theta , \delta \omega )$-parameter space. For each data point the maxima of one time series of one laser are counted and color coded in blue. In the locked regions the phase difference of the electric fields of the lasers is shown by the pink and yellow color code. From (**a**) to (**d**) the polarization lifetime $T_2$ increases. Other parameters are $\Delta \omega =3$, $p=2$, $K=0.1$, $\tau =10$, $T=392$.

Figure 6 shows two-dimensional bifurcation diagrams for which the coupling phase $\theta$ and the detuning $\delta \omega$ are varied. If the lasers stabilize on a CLM (locking region), the diagrams show the phase difference $\Delta \phi$ of the electric fields color coded with the yellow and pink color scale. Figure 6 a shows the results for a class-B laser while panels (b)–(d) show the results for $T_2 \in \{ 1,2,3 \}$, respectively. The class-B laser mainly differs from the class-C laser by its widespread exhibition of complex dynamics ($\ge 8$ maxima). For the class-C lasers, such areas are mainly replaced by harmonic oscillations (one maximum), i.e., the saddle-node bifurcations at the borders of the locking range are replaced by Hopf bifurcations. The extend and shape of the locking regions change dramatically with the polarization lifetime (Fig. 6 from top to bottom) and an optimal value of $T_2$ seems to exist. We investigate that in more detail in the next section.

The numeric results in Fig. 6 also nicely highlight the symmetries of our coupled laser system. Due to the $2\pi$-translational symmetry the results repeat and the system only needs to be investigated for $\theta \in [0,2\pi )$. The $\pi$-translational symmetry can be seen by focusing on the locked regions. If we perform a horizontal cut through the locking region along $\delta \omega =0$ the change from in-phase (pink shading) to anti-phase CLMs (orange shadings) always occurs after a phase shift of $\pi$. For every in-phase CLM at coupling phase $\theta _0$, a corresponding anti-phase CLM exists at phase $\theta _0+\pi$ solving Eqs. (1a)–(1c) equivalently. Therefore, also the stability of these in- and anti-phase CLMs coincides making the number of maxima or the locking regions $\pi$-periodic with respect to the coupling phase $\theta$. At last, also the reflection symmetry can be found in Fig. 6. It appears as a symmetry of the locking regions at the horizontal axis along $\delta \omega =0$.

## Locking range

The goal of this section is to identify a polarization lifetime that is best suited to attain large locking ranges. Thus, we define the locking range $\delta \omega _{lock}(\theta )$. It is the maximum detuning $\delta \omega >0$ for which the lasers lock onto a CLM for one specific coupling phase $\theta$ (see arrow in Fig. 6d for the definition). Negative detunings do not need to be considered since the locking regions are symmetrical. For some phases, the locking regions vanish completely, i.e., $\delta \omega _{lock}=0$. As can be seen in Fig. 6, $\delta \omega _{lock}$ also strongly depends on the polarization lifetime $T_2$. A monotonous correlation of the locking range to the polarization lifetime however can not be detected here, in contrast to a single class-C laser with feedback, which is stabilized by higher polarization lifetimes^12^.

**Figure 7:**
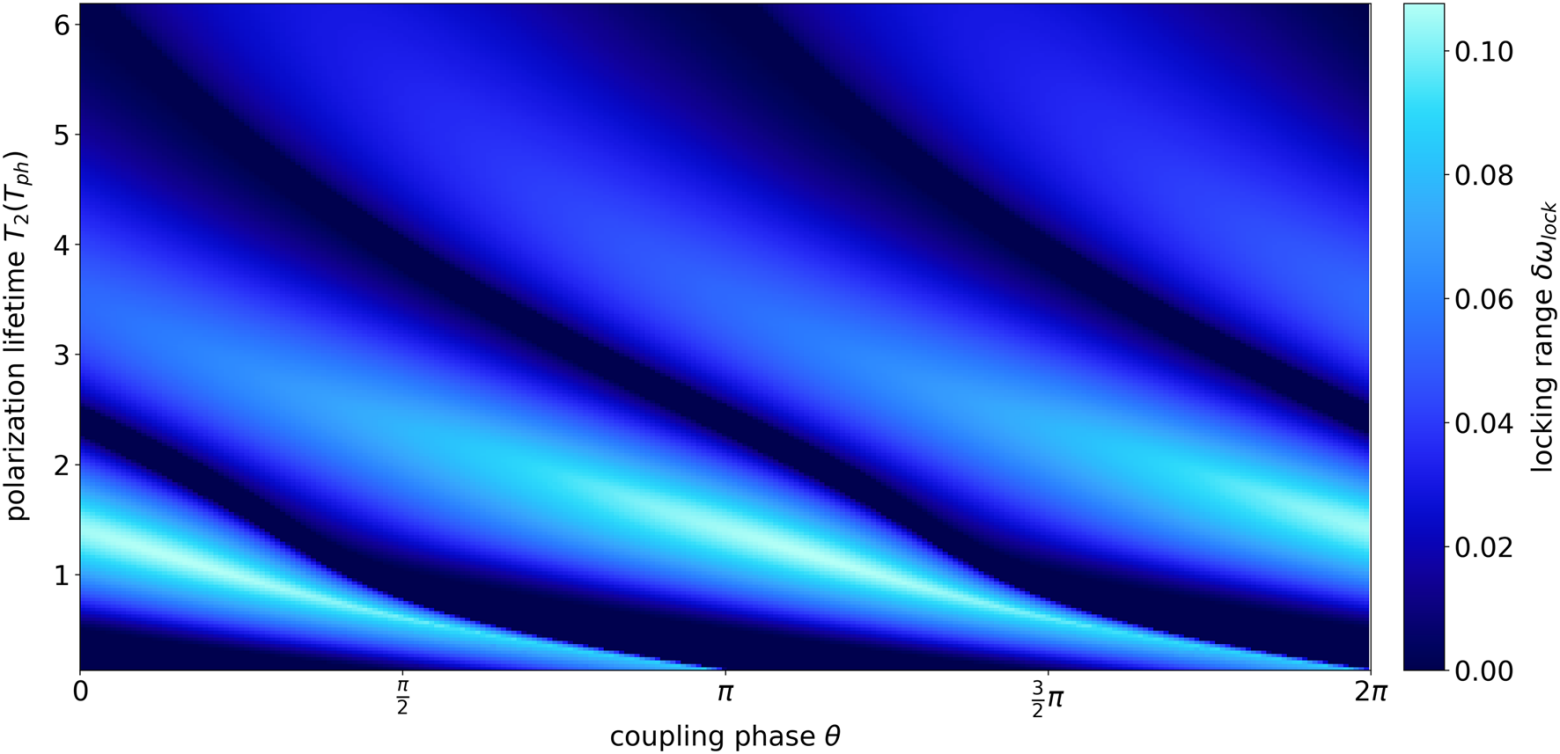
Color coded locking range $\delta \omega _{lock}$ as a function of the coupling phase $\theta$ and the polarization lifetime $T_2$ on the intervals $\left[ 0.1, 8 \right] _{T_2}$ and $\left[ 0, 2\pi \right] _{\theta }$. Other parameters are $\Delta \omega =3$, $p=2$, $K=0.1$, $\tau =10$.

To get a more comprehensive insight, Fig. 7 shows the locking range $\delta \omega _{lock}$ plotted color coded as a function of the coupling phase $\theta$ and the polarization lifetime $T_2$. Every horizontal line of the diagram holds the same information about the locking range as one sub-figure in Fig. 6. Presenting the situation continuously with respect to $T_2$ shows that the locking range $\delta \omega _{lock}$ does not vary monotonously with $T_2$ for any given coupling phase $\theta$. In fact, on the interval investigated here, there are at least two disjoint partial intervals for which the locking range $\delta \omega _{lock}$ vanishes.

We furthermore define the maximum locking range $\delta \omega _{max}^{\theta }$ as

9 $$\begin{aligned} \delta \omega _{max}^{\theta }(T_2) = \max _{\theta \in [0,2\pi )} \delta \omega _{lock}(\theta ,T_2). \end{aligned}$$

**Figure 8:**
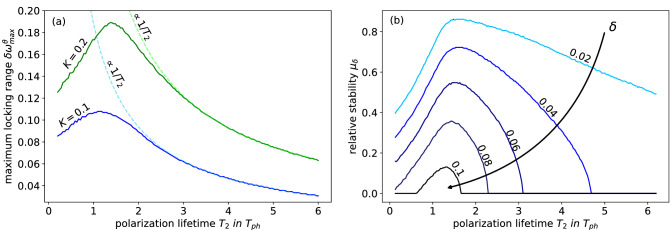
(**a**) Maximum locking range $\delta \omega _{lock}^{\theta }$, i.e., the largest locking range $\delta \omega _{lock}$ on the interval $[0,2\pi )_{\theta }$, as a function of the polarization lifetime $T_2$. The dashed lines are proportional to $1/T_2$. Results are presented for $K=0.1$ (blue) and $K=0.2$ (green). (**b**) Relative locking stability $\mu _{\delta }$ defined in Eq. (10) as a function of the polarization lifetime $T_2$ for different $\delta$ (black line). Other parameters are $\Delta \omega =3$, $p=2$, $K=0.1$, $\tau =10$ and $T=392$.

This maximally achievable locking range for the given coupling strength $K=0.1$ is depicted as a function of the polarization lifetime $T_2$ in Fig. 8a for coupling strengths of $K=0.1$ and $K=0.2$. For both coupling strengths, the maximum locking range $\delta \omega _{max}^{\theta }$ increases until it reaches its maximum at $T_2\approx 1.06$ for $K=0.1$ or $T_2\approx 1.39$ for $K=0.2$. Afterwards, it decreases with $\delta \omega _{max}^{\theta } \propto 1/T_2$ for $T_2 > 1.06$ or for $T_2 > 1.39$ (see dashed lines in Fig. 8a). The decrease of the maximum locking range correlates with the decrease of the gain spectrum (as its width scales with $1/T_2$) and consequently with a narrower frequency spectrum of possible CLMs (see “Compound laser modes (CLMs)” section). The increase of the maximum locking range for $T_2 <1.06$ on the other hand can be explained by the rising dynamical impact of the polarization lifetime and the additional degree of freedom in phase space compared to the class-B case. This additional dynamical degree of freedom also leads to a maximum around $T_2=1$ for the damping of the relaxation oscillations of the solitary laser (see Fig. 2). Both effects combined nicely explain the occurrence of an optimal value for $T_2$ as seen in Fig. 8a. Further, the maximum locking range $\delta \omega _{max}^{\theta }$ increases with the coupling strength *K* as shown in the plot. In particular, we found that it increases linearly for small coupling strengths below $K\approx 0.15$.

A second approach to characterize the locking behavior is by defining the relative stability $\mu _{\delta }$, which represents the relative share of coupling phases on the interval $[0,2\pi )_{\theta }$ for which the locking range exceeds a certain threshold $\delta$

10 $$\begin{aligned} \mu _{\delta }(T_2) = \int _0^{2\pi } \Theta (\delta \omega_{lock} (\theta , T_2)-\delta )d\theta. \end{aligned}$$

$\Theta$ denotes the Heavyside function. The relative stability gives further insights on how stable a given polarization lifetime $T_2$ is regarding all coupling phases. Figure 8b shows the relative stability $\mu _{\delta }$ for several thresholds $\delta$ as a function of the polarization lifetime $T_2$. Trivially, the relative stability increases if the threshold $\delta$, that has to be exceeded, decreases. For all shown $\delta$, the curves have maxima on the interval $[1,2]_{T_2}$. Hence, the polarization lifetime $T_2$ that exhibits the optimal maximum locking range $\delta \omega _{max}^{\theta }$ approximately matches the polarization lifetime that shows the biggest share $\mu _{\delta }$ of of high locking ranges. Both quantities together provide a comprehensive view of the locking behavior of the coupled laser system and show that optimal locking is achieved for polarization lifetimes of the order of the photon lifetime.

## Conclusion

We have investigated the locking and synchronization behavior of two coupled class-C lasers as a paradigmatic example of nanolasers with a small photon lifetime. An emphasis was put on the impact of the polarization lifetime, the frequency detuning, and the coupling phase. To approach the locking structure of the delay-coupled laser system, we have determined the compound laser modes, which are the fundamental steady-state solutions of the system, investigated the symmetries of the system, and performed a bifurcation analysis of the underlying dynamics. The polarization lifetime $T_2$ was found to decisively influence the locking range of the lasers. Interestingly a value of $T_2$ can be found where the locking range as well as the relative stability is optimal. The value depends on the photon lifetime within the cavity. Thus we can predict nanolasers to show best synchronization properties for the case where the photon lifetime is equal to the polarization lifetime for the case of weak coupling and about two third of the polarization lifetime for stronger coupling.

## Supplementary Information

Supplementary Video S1.
